# Two-Dimensional Image-Based Screening Tool for Infants with Positional Cranial Deformities: A Machine Learning Approach

**DOI:** 10.3390/diagnostics10070495

**Published:** 2020-07-19

**Authors:** Cecilia A. Callejas Pastor, Il-Young Jung, Shinhye Seo, Soon Bin Kwon, Yunseo Ku, Jayoung Choi

**Affiliations:** 1 Department of Biomedical Engineering, Chungnam National University College of Medicine, Daejeon 35015, Korea; cecilia.callejas.pastor@gmail.com (C.A.C.P.); seosh26@gmail.com (S.S.); 2 Department of Rehabilitation Medicine, Chungnam National University College of Medicine, Daejeon 35015, Korea; 102onez@hanmail.net; 3 Interdisciplinary Program in Bioengineering, Graduate School, Seoul National University, Seoul 03080, Korea; soonbin@melab.snu.ac.kr

**Keywords:** plagiocephaly, brachycephaly, positional cranial deformities, cephalic ratio, cranial vault asymmetry index, machine learning

## Abstract

Positional cranial deformities are relatively common conditions, characterized by asymmetry and changes in skull shape. Although three-dimensional (3D) scanning is the gold standard for diagnosing such deformities, it requires expensive laser scanners and skilled maneuvering. We therefore developed an inexpensive, fast, and convenient screening method to classify cranial deformities in infants, based on single two-dimensional vertex cranial images. In total, 174 measurements from 80 subjects were recorded. Our screening software performs image processing and machine learning-based estimation related to the deformity indices of the cranial ratio (CR) and cranial vault asymmetry index (CVAI) to determine the severity levels of brachycephaly and plagiocephaly. For performance evaluations, the estimated CR and CVAI values were compared to the reference data obtained using a 3D cranial scanner. The CR and CVAI correlation coefficients obtained via support vector regression were 0.85 and 0.89, respectively. When the trained model was evaluated using the unseen test data for the three CR and three CVAI classes, an 86.7% classification accuracy of the proposed method was obtained for both brachycephaly and plagiocephaly. The results showed that our method for screening cranial deformities in infants could aid clinical evaluations and parental monitoring of the progression of deformities at home.

## 1. Introduction

Positional cranial deformities occur when an external force or pressure produces asymmetry and flattening of the shape of the skull. A marked increase in the incidence of positional cranial deformities was also noted after the “Back to Sleep” campaign that was designed to identify these incidents and prevent sudden infant deaths. Therefore, currently, positional cranial deformity is a relatively common problem faced by parents and physicians. Specifically, in the first few months, cranial deformities in infants have more prevalence on the occipital bone, manifested as unilateral or bilateral flattening [1,2]. According to a prior study, the prevalence of positional skull deformities at the age of 6 weeks is 16% and decreases to 3.3% by the age of 24 months [3]. Another prevalence investigation that reviewed 18 different studies reported a similar trend. It also showed that the prevalence is age-dependent, can be as high as 22.1% at 7 weeks of age, and tended to decrease to as little as 3.3% at 2 years [4].

These cranial deformities are diagnosed and quantified based on the following clinical features: (1) deformational brachycephaly is a bilateral symmetric occipital flattening with a parietal widening induced as a compensatory reaction characterized by an increase in the cranial width-to-length ratio; (2) deformational plagiocephaly is a single-sided oblique flattening on the back of the head and comparable to a trapezoidal shape. This asymmetric growth is often accompanied by a frontal face asymmetry [1,5]. Sociodemographic and socioeconomic factors, including young parents or low-educational status, and obstetric factors such as multiparity, delivery complications, or prematurity, increase the risk of positional head deformities. Moreover, infant care, such as head positioning during sleeping, is strongly related to the positional head deformity [2,4].

For treating children with deformational plagiocephaly and brachycephaly, different interventions, such as repositioning, physical therapy, or cranial orthosis, are applied depending on the characteristics, severity, and age [4,5,6]. However, regardless of the deformation severity, the positional head deformity can be significantly improved when the appropriate treatment begins at an early stage, especially before reaching the age of 6 months [6,7,8]. This is why early diagnosis is highly recommended in this field. In addition to the vast majority of treatments in the clinic, continuous monitoring and positional management by parents at home are also crucial for the improvement of head deformities [9].

In the clinic, the standard diagnostic procedure uses a sliding caliper or a craniometer as a tool for the manual measurement of the cranial deformity; this method is inconvenient and possibly inaccurate [10,11]. Although three-dimensional (3D) cranial scans are considered the gold standard in diagnosis and can provide more accurate and reliable information, they require an expensive laser scanner with a head cap and a skilled operator [12,13,14]. Other 3D imaging techniques, such as stereophotogrammetry and procedures involving structured light patterns, are becoming increasingly important for the diagnosis of skull abnormalities [15].

In this study, we aimed to develop a novel screening tool for evaluating the level of brachycephaly and plagiocephaly that can support a physician’s assessment procedure in the clinic as well as the monitoring conducted by parents at home. Thus, we investigated two-dimensional (2D) image-based machine learning models to estimate deformity evaluation indices including image processing and feature extraction. We aimed to implement the developed model as a software tool to allow users to perform follow-up tracking of the progression of head deformities.

## 2. Materials and Methods 

### 2.1. Participants and Data Collection

This retrospective study was approved by the Institutional Review Board of the Chungnam National University Hospital (Institutional Review Board no. 2019-10-121, approved on 6 December 2019). Data collection was performed from 2017 to 2019, and the database contained a total of 174 measurements obtained from 80 subjects (30 females and 50 males between 4 and 14 months of age). The number of measurements recorded per patient varied depending on each case. Some patients underwent several clinical evaluations at different points in time to track the progress of the head deformity. The average number (±standard deviation) of measurements for each subject was 2.2 (±1.3), and the maximum number of assessments was seven. In all the measurements, an operator scanned the patient’s head, which was covered with a cap. Scanning was conducted in various directions to digitize the shape of the cranial vertex and lasted for a few minutes. Anthropometric parameters were calculated with a dedicated software that was part of the 3D body scanner system (TechMed 3D Inc., Québec, Canada). The cephalic or cranial ratio (CR) and the cranial vault asymmetry index (CVAI) were automatically calculated at the cross-sectional plane by passing a third point (plane 3) between the planes passing through the sellion and tragia (plane 0) and the vertex of the head (plane 10). The reference CR and CVAI were both obtained using the same 3D scanner. After this procedure, a 2D vertex image of the subject’s head, covered by the very thin and stretchable white cap, was acquired using a commercial digital camera (IXUS 185, Canon Inc., Tokyo, Japan). We manually aligned a camera as parallel as possible to the subject’s head.

### 2.2. Diagnostic Indices for Head Deformities

**Figure 1:**
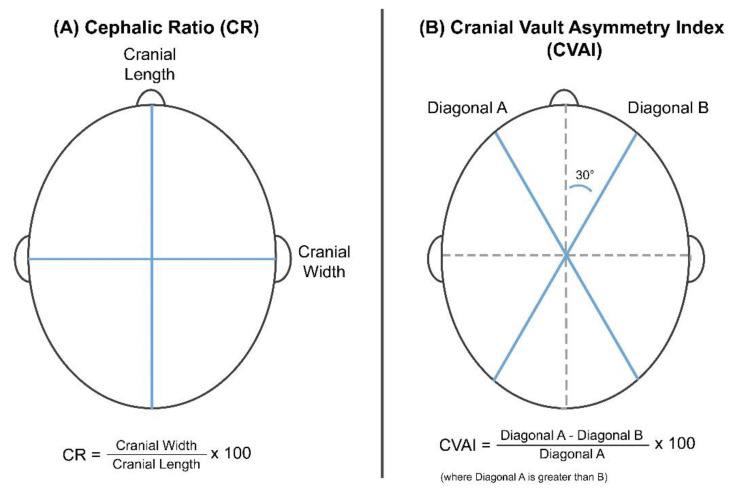
Reference parameters and formulas used to calculate the (**A**) cranial ratio (CR) and (**B**) cranial vault asymmetry index (CVAI) indices.

The 3D scanner obtains the anthropometric measurement information, including the length, width, and diagonals A and B, as shown in Figure 1A [12,16]. The CR is defined as a percentage of the horizontal width to the vertical length of the head.

(1) $$CR=\frac{Cranial\;Width}{Cranial\;Length}\times 100$$

Given that the CR represents the width/length relationship, it is used as a severity index for the brachycephaly. The severity is usually classified into four levels depending on CR values: normal, mild, moderate, and severe deformations. Higher CR scores indicate more severe brachycephaly states (Table 1A) [17].

The CVAI represents the asymmetry of the skull, as shown in Figure 1B.

(2) $$CVAI=\frac{Diagonal\;A-Diagonal\;B}{Diagonal\;A}\times 100$$

where diagonal A is greater than diagonal B. This index stratifies plagiocephaly into five severity levels; higher CVAI scores indicate severe asymmetry (Table 1B) [18,19,20].

### 2.3. Image Processing

**Figure 2:**
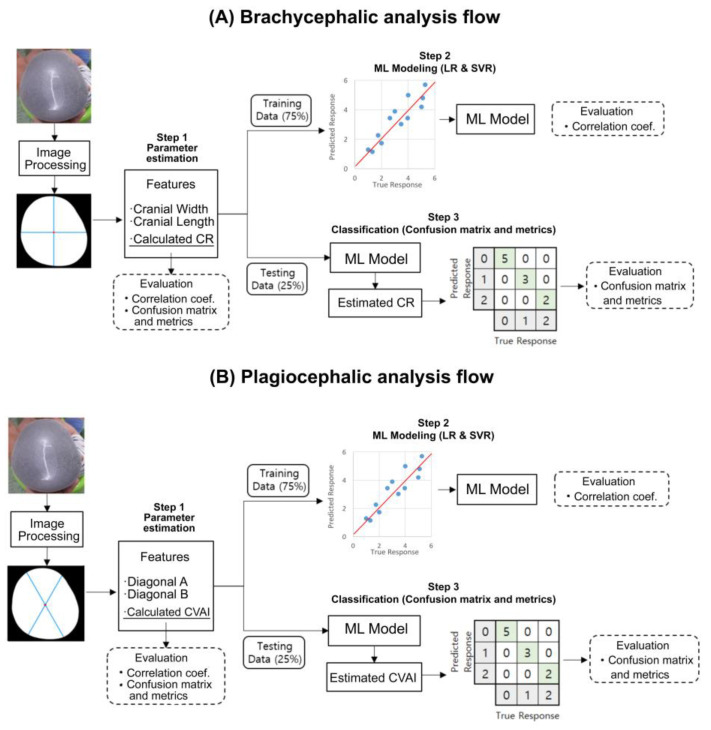
Flow diagram of our novel approach implemented as a head deformity screening tool and used for (**A**) brachycephalic and (**B**) plagiocephalic analyses. Image segmentation, parameter estimation, and machine learning (ML) modeling were executed with the training set (75%), while severity level classification and metrics calculation were executed with the test dataset (25 In the confusion matrices, grey squares indicate actual and predicted severities of head deformity while diagonal green squares indicate correct classifications.

**Figure 3:**
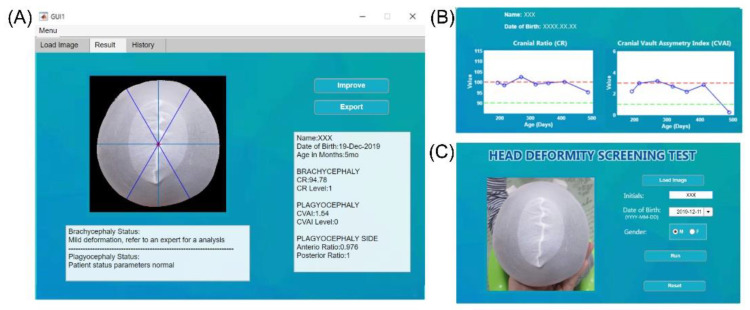
Graphical user interface (GUI) of the developed tool. Panel (**A**) is where the user can upload an image and insert the patient’s information. Panel (**B**) lists the history, wherein the name of a specific patient can be selected and the progress of the deformity can be evaluated according to the date of the assessment. The solid blue line indicates evaluation results for different points in time, and the two dotted lines indicate the criteria for normal (green) and severe (red) head deformity. Panel (**C**) shows the results of the analysis and the guide provided to the user.

Figure 2 shows the overall development flow, including image processing, feature extraction, machine learning model training, and final performance testing. All processes were conducted using MATLAB (version 2019b, Mathworks, Natick, MA, USA). For image segmentation, the grab-cut method was used to associate pixels of similar colors, and then the image was segmented into foreground and background sections to reconstruct the shape of the subject’s head. Erosion was then applied to eliminate noise, followed by closing. The edges were also refined. Figure 3 shows the developed graphic user interface (GUI) software that provides an additional option for the manual selection of the mis-segmented points from the selected area.

### 2.4. Feature Extraction

After the segmentation process, the features for CR and CVAI calculations were extracted. First, the center point of the segmented image was calculated using the centroid function for a polyshaped object. A horizontal line and a right-angled vertical line were drawn through this point to the polyshaped boundary that constituted the outline of the subject’s head. The lengths of these two lines were considered as the cranial width and length, respectively. Subsequently, two lines, oriented at 30° with respect to the vertical axis from the left and right directions and intersecting the center of the segmented image, were drawn for the calculation of the diagonals A and B. Lastly, the CR and CVAI values were calculated based on Equations (1) and (2), respectively.

### 2.5. Machine Learning Modeling

For modeling the relationship between the calculated values and 3D scanned reference indices, the data were divided into training and test sets at a 75% (129 images) to 25% (45 images) ratio. The distributions of CR and CVAI classes in the training and test sets were balanced, but the data selection was randomly performed in all the CR and CVAI classes. Two different regression models were applied, namely multiple linear regression (LR) and support vector regression (SVR), for the training and test datasets. The SVR is a supervised machine learning model that projects nonlinear separable data into another dimensional space (usually higher) by applying a kernel function. The purpose of training is to determine the hyperplane that maximizes the margin of tolerance, which can optimally separate continuous data based on the labeled training data. Two separate models were developed to estimate CR and CVAI. When estimating CR, the selected input features were the estimated cranial width, estimated cranial length, and calculated CR based on the reference formula. In CVAI estimation, the selected input features were the estimated diagonal A, estimated diagonal B, and calculated CVAI by the reference formula.

### 2.6. Model Evaluation

First, correlation coefficients between the estimated CR and CVAI values and the 3D scanned reference data were used to investigate the relationship for continuous indices. Second, confusion matrices were generated as classification tools to evaluate the performance. Both the CR and CVAI were classified into three levels of severity: normal, moderate, and severe. However, owing to the small number of patients with extreme deformities, the most severe group was merged with the one-step lower group for CR and CVAI in this study. For the CVAI, the mild group was also merged with the moderate I group, as shown in Table 2. This study aimed to develop a convenient and supportive screening tool to detect head deformities, rather than an accurate diagnostic device. The accuracy that is exploited as the metric parameter is calculated according to

(3) $$Accuracy=\frac{TP+TN}{TP+TN+FP+FN}$$

where true positives (TP) and true negatives (TN) represent the number of correct positive and negative predictions, respectively, and false positives (FP) and false negatives (FN) are the incorrect positive and negative predictions, respectively [21]. For the second metric, the F1 score represents the harmonic mean of precision (TP/(TP+FP)) and recall (TP/(TP+FN)), and is calculated as [22]

(4) $$F1\;Score=2\times \frac{precision\times recall}{precision+recall}$$

In this study, a weighted F1 score that assigns a weight to each class based on its data content was used because the multiclasses were not well balanced [23,24].

This study included three evaluation steps. First, we observed the linear relationship between the values calculated from the extracted features and references for both CR and CVAI. The classification performance was also evaluated without any training procedure. The above correlation coefficients and classification performance were considered as the reference points that the proposed model should outperform. Second, the correlation coefficients were obtained from the LR and SVR modeling of the training dataset. Third, accuracy and the weighted F1 score of the classification performance with the estimated CR and CVAI from the LR and SVR models were evaluated in the unseen test dataset.

## 3. Results

**Figure 4:**
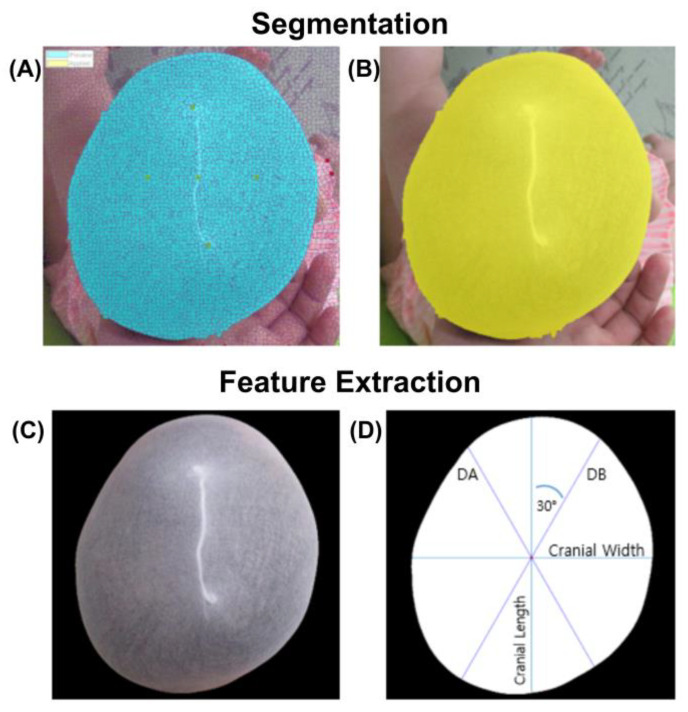
Image segmentation and feature extraction process. (**A**) Pixel clustering and association used for segmentation (blue) (**B**) Boundary delimitated segment (yellow). (**C**) Removal of small regions on the edge of the image and cleaning of the boundary. (**D**) Parameter setup and estimation: cranial length, cranial width, diagonal A (DA) and diagonal B (DB).

All images were visually inspected after image processing. Only seven images required a manual selection option. It took only 1–2 min to execute all the procedures. After the execution of the manual procedure, all the images were included in the study. Figure 4 shows a typical outcome after the execution of all the image processing operations.

### 3.1. Brachycephalic Analyses

**Figure 5:**
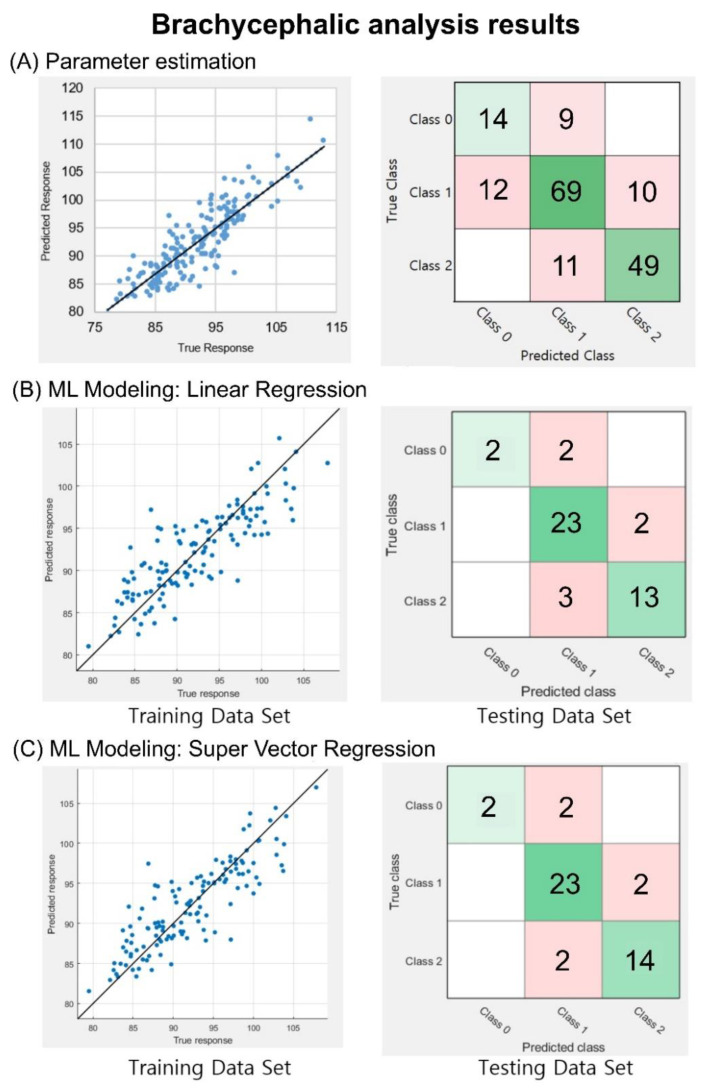
Brachycephalic analysis outcomes. (**A**) Correlation analysis and classification analysis using the information from the 174 database entries. CR index correlation graph and equation that corresponds to linear regression (LR) (correlation coefficient R = 0.86). CR machine learning modeling approaches, the correlation between training dataset, and reference and testing datasets classified on a confusion matrix. (**B**) LR model correlation coefficient of 0.84 and a classification outcome based on three severity levels. In this case, 38 out of 45 images were correctly classified. (**C**) SVR model correlation coefficient of 0.85 and a classification based on three severity levels. In total, 39 of the 45 images were correctly classified. In the confusion matrices, diagonal green squares indicate correct classifications while off-diagonal red squares indicate incorrect classifications. The color intensity of each green square is proportional to the cell value.

The correlation coefficient between the calculated CR and reference data was 0.86. For the classification of severity levels, an accuracy of 75.9% and a weighted F1 score of 0.76 were obtained, as shown in Figure 5A. In the training session of the CR values, the respective correlation coefficients between the estimated and reference data were 0.84 and 0.85 when the LR and SVR were applied, respectively, as shown in the left panels of Figure 5B,C. In the test session of the trained models, the LR model showed an accuracy of 84.4% and a weighted F1 score of 0.84, while the SVR model showed an accuracy of 86.7% and a weighted F1 score of 0.86. This result indicates that 39 of the 45 testing set data images were successfully classified, as shown in the right panels of Figure 5B,C.

### 3.2. Plagiocephalic Analyses

**Figure 6:**
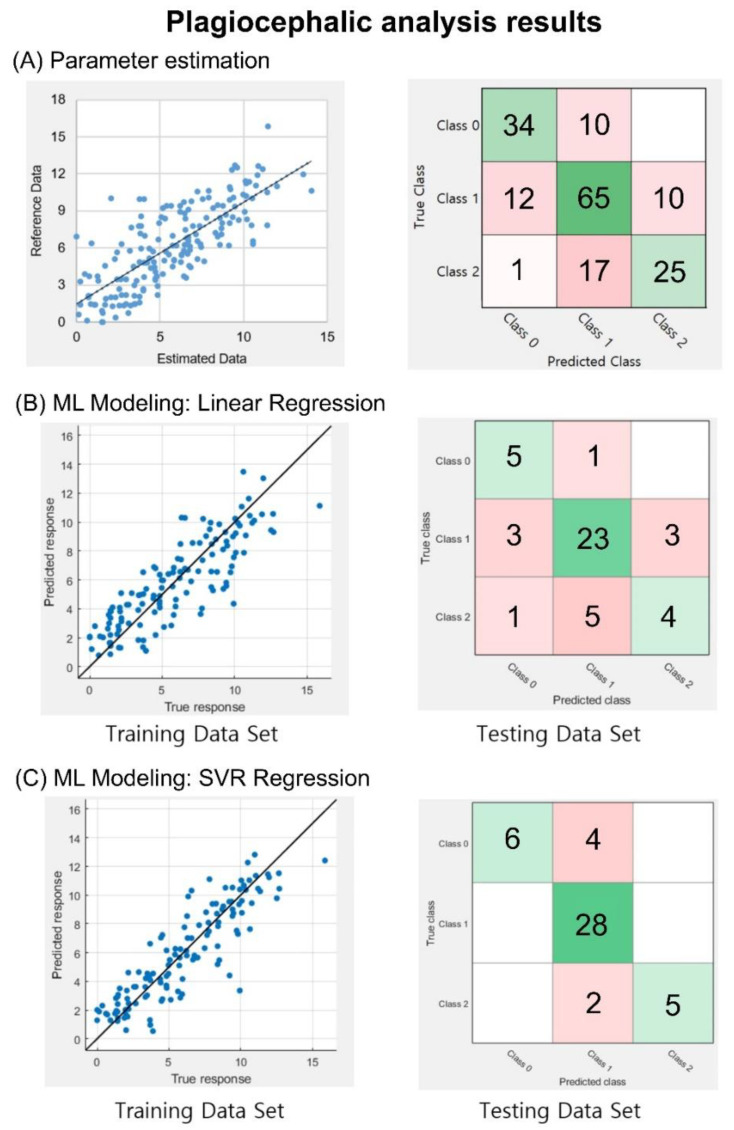
Plagiocephaly analysis outcomes. (**A**) Correlation and classification analysis using information from 174 database entries. CVAI index graph with a correlation coefficient of R = 0.77. CVAI machine learning modeling approaches, the correlation between training, reference, and testing datasets classified based on a confusion matrix. (**B**) LR model, with a correlation coefficient of 0.84 and a confusion matrix with 32 of the 45 images correctly classified. (**C**) SVR model, with a correlation coefficient of 0.89, and the correct classification of 39 of the 45 images. In the confusion matrices, diagonal green squares indicate correct classifications while off-diagonal red squares indicate incorrect classifications. The color intensity of each green square is proportional to the cell value.

Similar to the brachycephalic analysis, the correlation coefficient between the calculated CVAI and reference data was 0.77. In addition, for the classification of severity levels, an accuracy of 71.3% and a weighted F1 score of 0.71 were obtained, as shown in Figure 6A. In the training session for the CVAI values, as shown in the left panels of Figure 6B,C, the correlation coefficients between the estimated and reference data were 0.84 and 0.89 with LR and SVR, respectively. In the test session of the trained models, the accuracy was 71.1%, and the weighted F1 score was 0.67 for the LR model, while the SVR model yielded an accuracy of 86.7% and a weighted F1 score of 0.86. This result indicates that 39 of the 45 images in the testing set data were successfully classified, as shown in the right panels of Figure 6B,C.

## 4. Discussion

In this study, we proposed a simple screening method that required only 2D cranial images to assess cranial deformities. This method can detect cranial deformities early and also monitor the effectiveness of interventions. 

In image processing, seven images required manual procedures owing to the similar colors of objects such as clothes or the ground near the head area. Given that the developed software used 2D images, it is recommended that the colors of the patient’s clothing and the image backgrounds differ from the hair cap or hair color to eliminate the need to use the hair cap and to achieve accurate segmentation. We included a manual selection option to compensate for this issue, but full, automated segmentation could be achieved by avoiding noise in the image. 

When the diagnostic indices of head deformities were calculated, the line that represents the cranial width should be located at the position of the patient’s ears. Unfortunately, images showing ears are very rare because infants have tiny ears that are often covered by hair caps. Moreover, when images are acquired, the exact alignment of the camera position with the infant’s cranial vertex is not guaranteed. Accordingly, this can introduce an error in the image acquisition process. However, providing a user guide to request the alignment of a camera as parallel as possible to the patient’s head could be an alternative to minimize this error. We aimed to use a machine learning approach because we hypothesized that even if the anthropometrical positions of ears and nose according to the size and shape of the crane varied slightly from one person to another, these parameters were trainable. The accuracy improvements achieved by applying machine learning model were 10.8% and 15.4% for CR and CVAI, respectively, in terms of the severity classification. These results could support the notion that the machine learning approach is feasible when 2D images are used. While other methods require external equipment, such as several markers or peripheral measurement tools [12,25], our system uses only one digital camera, such as the camera installed on smartphones, and performs segmentation and screening automatically. In the future, our screening model can be improved using an additional 2D image and 3D reference data. When a sufficiently large dataset is acquired, the deep learning algorithm, such as the convolution neural networks that use 2D images as model inputs, can be applied. Additionally, datasets of sufficient size could prevent the merging of head deformity levels, which lowered the sensitivity of our screening tool in this study. The use of time-of-flight (ToF) sensors installed on some smartphones can add depth information in image segmentation, making image processing more precise and accurate. The 3D images acquired using smartphones can also provide useful information, such as cranial volume, for assessment. Our current personal computer-based software can be implemented on mobile devices, which would allow beneficial access to the image data at home. We can use a vertex skull image and frontal and lateral images to expand the face deformities related to the head deformities and to improve accuracy with reference to the features extracted from complex computerized shape analysis tools [26]. Our study was validated only internally and retrospectively. For further prospective validation of the model for prospective use in clinical practice, it is appropriate to validate it with external data, such as image data acquired from home. Further studies employing images without a cover cap should be conducted to improve convenience for use at home. Another limitation of this method is its limited role in the etiology workup of skull deformities such as lambdoid craniosynostosis. Therefore, this screening tool should only be used to assess and monitor the degree of positional cranial deformation. Moreover, a relatively low accuracy in the normal class was observed, owing to the lack of sufficient data, leading to an unbalanced amount of data between classes. Further training with more data on normal subjects is warranted in order to improve model performance. We proposed an inexpensive, fast, and convenient screening method that required only a cranial vertex image to classify the severity of brachycephaly and plagiocephaly. Our screening tool performs multiple procedures, including image segmentation, feature calculation, machine learning-based CR and CVAI estimation, and severity level classification of head deformities. When our results were compared with those obtained from a 3D scanner system, the correlation coefficients were 0.85 and 0.89 for CR and CVAI, respectively. Moreover, the classification accuracy of our proposed model was 86.7% for both brachycephaly and plagiocephaly. These results indicate that the proposed tool is suitable for screening purposes in the clinic and at home.

## Figures and Tables

**Table 1:** Head positional deformity severity scales.

	**(A) Cranial Ratio**	
**Brachycephaly Severity**	**Level**	**Value**
Normal	0	75.0–84.9
Mild Brachycephaly	1	85.0–94.9
Moderate Brachycephaly	2	95.0–104.9
Severe Brachycephaly	3	≥105.0
	**(B) Cranial Vault Asymmetry Index**	
Plagiocephaly Severity	Level	Value
Normal	0	≤3.49
Mild Plagiocephaly	1	3.50–6.24
Moderate Plagiocephaly I	2	6.25–8.74
Moderate Plagiocephaly II	3	8.75–10.99
Severe Plagiocephaly	4	≥11.00

**Table 2:** Head positional deformity merged with severity scales.

	**(A) Cranial Ratio**		
**Brachycephaly Severity**	**Class**	**CR Value**	**No. Cases**
Normal	0 (Level 0))	75.0–84.9	23
Moderate Brachycephaly	1 (Level 1)	85.0–94.9	91
Severe Brachycephaly	2 (Level 2 & 3)	≥95.0	60
Total			174
	**(B) Cranial Vault Asymmetry Index**		
**Plagiocephaly Severity**	**Class**	**CVAI Value**	**No. Cases**
Normal	0 (Level 0)	≤3.49	43
Moderate Plagiocephaly	1 (Level 1 & 2)	3.50–8.74	88
Severe Plagiocephaly	2 (Level 3 & 4)	≥8.75	43
Total			174
